# Reference genes for normalising gene expression data in collagenase-induced rat intracerebral haemorrhage

**DOI:** 10.1186/1471-2199-11-7

**Published:** 2010-01-20

**Authors:** Naomi L Cook, Timothy J Kleinig, Corinna van den Heuvel, Robert Vink

**Affiliations:** 1 Discipline of Anatomy and Pathology, School of Medical Sciences, The University of Adelaide, Adelaide SA 5005, Australia

## Abstract

**Background:**

The mechanisms of brain injury following intracerebral haemorrhage (ICH) are incompletely understood. Gene expression studies using quantitative real-time RT-PCR following ICH have increased our understanding of these mechanisms, however the inconsistent results observed may be related to inappropriate reference gene selection. Reference genes should be stably expressed across different experimental conditions, however, transcript levels of common reference genes have been shown to vary considerably. Reference gene panels have therefore been proposed to overcome this potential confounder.

**Results:**

The present study evaluated the stability of seven candidate reference genes in the striatum and overlying cortex of collagenase-induced ICH in rodents at survival times of 5 and 24 hours. Transcript levels of the candidate reference genes were quantified and ranked in order of stability using geNorm. When our gene of interest, transient receptor potential melastatin 2 (TRPM2), was normalised against each reference gene individually, TRPM2 mRNA levels were highly variable. When normalised to the four most stable reference genes selected for accurate normalisation of data, we found no significant difference between ICH and vehicle rats.

**Conclusion:**

The panel of reference genes identified in the present study will enable more accurate normalisation of gene expression data in the acute phase of experimental ICH.

## Background

Intracerebral haemorrhage (ICH) accounts for around 10 - 15% of all strokes worldwide, with a higher proportion in Asian countries. Compared with ischaemic stroke, it causes disproportionate mortality and morbidity [1] and research efforts to understand its pathophysiology are accordingly of high importance [2]. Brain injury following ICH is a dynamic process, due both to rebleeding and to downstream injury pathways activated by the blood clot [3]. However, no therapies addressing these secondary injury pathways have been proven beneficial.

One approach to understanding these injury pathways is to study gene expression in animal models using quantitative real-time reverse transcription polymerase chain reaction (real-time RT-PCR). Real-time RT-PCR is the method of choice for quantifying mRNA transcripts due to its wider dynamic range of quantification, higher sensitivity and precision, and a decreased risk of contamination compared to gel-based PCR [4-6]. Data obtained from real-time RT-PCR assays require a reliable method of normalisation to correct for such factors as differences in quantity and quality of RNA samples and for efficiency of the reverse transcription reaction [7,8]. Several normalisation strategies exist, the most common being the use of one or more endogenously expressed reference genes [9]. A reference gene should be expressed at a stable level in different tissue types and be unaffected by the experimental condition under investigation [10]. However, several of the most commonly used reference genes, such as glyceraldehyde-3-phosphate dehydrogenase (GAPDH) and β-actin, have been shown to have variable expression patterns and thus to be unsuitable for normalising real-time RT-PCR data in certain conditions [11-14]. The use of an inappropriate reference gene for normalisation may lead to incorrect data interpretation [15]. In contrast, normalisation to the geometric mean of the expression of multiple reference genes [16] is considered to be a reliable and conservative approach [17].

Previous studies employing quantitative real-time RT-PCR in rat ICH models have utilised a single reference gene for normalisation, usually without including a reference gene validation protocol [18-22]. Therefore, as an example, we demonstrate the impact of different reference gene selection methods on determining the transcript levels of transient receptor potential melastatin 2 (TRPM2) channels following ICH. TRPM2 consists of a calcium-permeable channel fused to a protein kinase domain [23], and is highly expressed in the brain and immune cells [24,25]. It has been implicated in cell death pathways relating to oxidative stress [26,27] and ischaemia [28] and its mRNA level has been shown to increase in a time-dependent manner following transient middle cerebral artery occlusion in rats [29]. No studies to date have quantified the mRNA level of TRPM2 following ICH.

We aimed to identify the most appropriate reference genes to normalise real-time RT-PCR data in the collagenase model of ICH in rats. Accordingly, real-time RT-PCR was used to measure transcript levels of seven reference genes: GAPDH, β-2-microglobulin (B2MG), RNA Polymerase II (POL2R), TATA Box Binding Protein (TBP), hypoxanthine guanine phosphoribosyltransferase (HPRT), succinate dehydrogenase complex, subunit A (SDHA) and β-glucuronidase (GUSB), plus our gene of interest, TRPM2. We initially normalised TRPM2 data to individual reference genes using the relative standard curve method [30]. Next, a reference gene validation study was conducted using the geNorm application [16] to rank reference genes in order of stability. Other software programs are also available to assess the stability of candidate reference genes, including Normfinder [31] and BestKeeper [32]. Although different algorithms are utilised in each application, studies have reported fairly consistent reference gene rankings between all three programs, particularly with regard to identifying the least stable genes [33-35]. However, we chose to use geNorm in the present study given that it also determines the number of reference genes required for accurate normalisation. The panel of stable reference genes identified by geNorm was then applied to normalise data regarding our gene of interest, TRPM2. The results presented herein will help to provide a guideline for selecting stable reference genes for normalising real-time RT-PCR data in future studies.

## Results

### RNA Concentration and Integrity

The concentration of extracted total RNA was quantified by measurement of the absorbance at 230 nm, 260 nm and 280 nm using a UV spectrophotometer. All samples had A260:A280 ratios of between 2.1 and 2.2, and A260:A230 ratios of between 2.2 and 2.6. RNA integrity was assessed using automated micro-capillary electrophoresis in the Agilent Bioanalyzer. An RNA Integrity Number (RIN) (reviewed in [36]) was assigned to each sample by the Agilent Bioanalyzer Expert 2100 software. All RIN values obtained were in the range of 8.6 - 9.4, representing high quality RNA with minimal degradation.

### Real-time RT-PCR

Primer pairs and reaction conditions were optimised using the standard cDNA pool prior to amplifying unknown cDNA samples. Melting curve analysis was consistent with a single reaction product for each gene, and product size was confirmed by 2% agarose gel electrophoresis stained with ethidium bromide and visualised with UV light (not shown). After validation of primer specificity, real-time PCR was carried out for unknown collagenase ICH and saline vehicle cDNA samples and serial dilutions from the cDNA pool. Reaction efficiencies were calculated automatically by the Corbett Rotor-Gene 6 software. The efficiency of all runs was between 95% and 105%. Minimum R^2 ^values of 0.985 were accepted for each run, however, most runs had R^2 ^values over 0.99.

### TRPM2 mRNA Level Normalised to Individual Reference Genes

**Figure 1:**
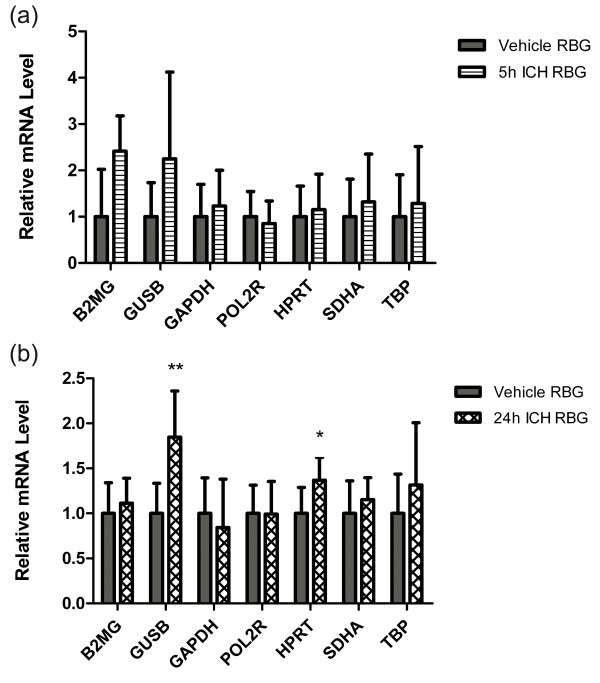
**Relative mRNA level of TRPM2 normalised to individual reference genes**. TRPM2 mRNA level at (A) 5 hours and (B) 24 hours in the perihematomal brain region (RBG) of collagenase-induced ICH rats compared to saline vehicles. Bars represent mean of triplicate measurements from 5 animals per group ± SEM. Single asterisk denotes statistical significance (*p *< 0.05) between 24 h ICH and vehicle rats; double asterisk denotes statistical significance (*p *< 0.01) between 24 h ICH and vehicle rats, as assessed by t-tests.

The relative standard curve method [30] was used to calculate TRPM2 mRNA level in the perihematomal region of ICH and vehicle rats with survival times of 5 h and 24 h, relative to each of the seven reference genes individually. Figure 1a shows TRPM2 mRNA level at 5 h post-ICH. Large variations were observed depending on which reference gene was used for normalisation. At the 24 hour time point, when TRPM2 data were normalised to GUSB only, a significant (*p *< 0.01) 1.85-fold increase in mean TRPM2 mRNA level was observed in the collagenase ICH rats compared to saline vehicles (Figure 1b). A significant (*p *< 0.05) 1.4-fold increase was found in TRPM2 mRNA level when data were normalised to HPRT only. When each of the other reference genes was used individually for normalisation, there were no significant differences. Given the discrepancy in these results and the large variation between samples, we proceeded with a reference gene evaluation study to determine the most stable reference genes in the collagenase model of ICH.

### Reference Gene Stability

The geNorm v3.5 application was used to determine the most stable reference genes out of the seven candidate genes tested. We were interested to see whether the stability of the different reference genes was influenced by the different survival times of the collagenase ICH rats (5 hours and 24 hours), and whether the injury itself could produce localised fluctuations in reference genes between the perihematomal (RBG) and matching contralateral (LBG) regions of the brain. Therefore, we used geNorm to ascertain the most stable reference genes in the following five groups: a collective group of all collagenase ICH and saline vehicle samples; 5 hour collagenase ICH and saline vehicle samples; 24 collagenase ICH and all saline vehicle samples; RBG only from all collagenase ICH and saline vehicle samples; LBG only from all collagenase ICH and saline vehicle samples. The most stable reference genes for each group plus recommended number of reference genes for accurate normalisation are summarised in Table 1.

**Table 1:** Ranking of candidate reference genes in order of stability as determined by geNorm.

All ICH and saline vehicle rats	5 hour ICH and saline vehicle rats	24 hour ICH and saline vehicle rats	RBG of all ICH and saline vehicle rats	LBG of all ICH and saline vehicle rats
B2MG*/GUSB*	GAPDH*/HPRT*	B2MG*/GUSB*	HPRT*/SDHA*	B2MG*/GUSB*
POL2R*	POL2R*	POL2R*	GAPDH*	POL2R*
GAPDH*	SDHA*	GAPDH	B2MG*	SDHA*
SDHA	B2MG	SDHA	GUSB*	GAPDH
HPRT	GUSB	HPRT	POL2R	HPRT
TBP	TBP	TBP	TBP	TBP

Ranking of candidate reference genes in order of stability as determined by geNorm. The most stable reference genes are listed at the top and the least stable at the bottom. * indicates number of reference genes required for accurate normalisation of real-time RT-PCR data, revealed by pairwise variation analysis. RBG and LBG refer to the perihematomal and the contralateral region, respectively, of collagenase ICH and vehicle rats.

In the combined group of all samples, B2MG and GUSB were found to be the most stable reference genes, while TBP was the least stable. Indeed, B2MG and GUSB were the two most stable reference genes in 3 out of the 5 groups tested, while TBP was ranked as the least stable gene in all 5 groups. Overall, HPRT displayed variable stability; it was ranked among the least stable two genes in 3 out of 5 groups, but conversely was among the top two stable genes in the other 2 groups. POL2R was never one of the two most stable genes in any group, however, it was consistently ranked as the third most stable reference gene in 4 out of 5 groups. SDHA and GAPDH exhibited reasonable stability across the different groups.

**Figure 2:**
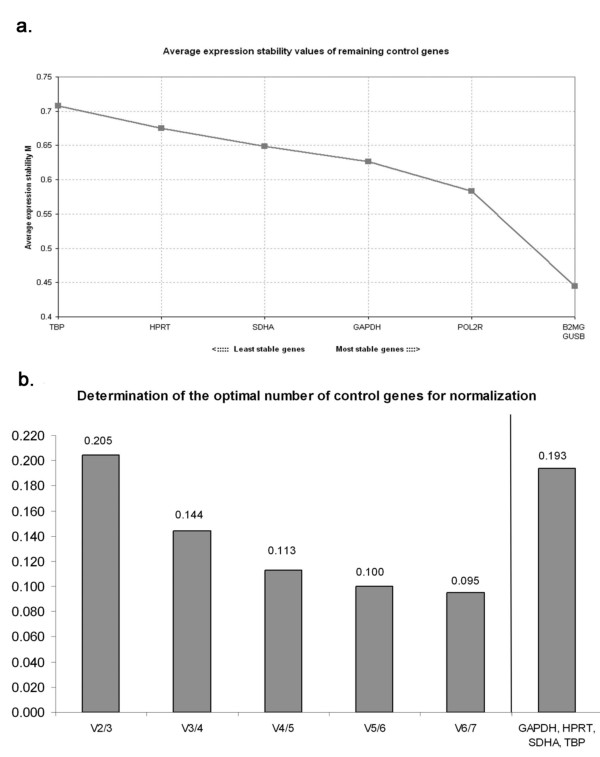
**Ranking of candidate reference genes in order of stability by geNorm**. (A) Expression stability values (*M*) of candidate reference genes in 5 h and 24 h collagenase-induced ICH and saline vehicle rats. geNorm ranks reference genes in order of least stable to most stable (left to right) by stepwise exclusion of the least stable gene. (B) Pairwise variation (V) analysis determines the optimal number of reference genes that should be used for accurate normalisation, with a threshold value of 0.15 [16]. In this case, the V_3/4 _step achieves the cut-off value, and therefore, the appropriate number of reference genes for accurate normalisation is four. The far right bar represents pairwise variation when the three most stable genes are removed from analysis and the stability of the remaining genes (GAPDH, HPRT, SDHA and TBP) are re-assessed. In this case the V threshold is not met.

Figure 2 shows geNorm output charts from the combined group of all collagenase-induced ICH and saline vehicle rats. All of the candidate reference genes had expression stability (*M*) values under the recommended cut-off value of 1.5 [16]. The highest *M *value obtained from all sample subsets tested was 0.867 (not shown). Figure 2a shows *M *values for each reference gene and ranks genes in order of stability from left to right. geNorm does not discriminate between the two most stable genes since it relies on pairwise correlations. In Figure 2b, pairwise variation (V) analysis determined that four reference genes were optimal for accurate normalisation in the collective group, indicated at the V_3/4 _step of the chart. The ideal value of V (y-axis) is recommended to be under 0.15 [16] and was achieved in all sample subsets. Given that all of our candidate reference genes had *M *values well below 1.5 and that the pairwise variation threshold was achieved even when all seven genes were used for normalisation (V_6/7 _step in Figure 2b), we determined whether even the least stable genes would serve as appropriate normalisation factors (i.e. meet the geNorm *M *and V thresholds) when the three most stable genes were removed from analysis. Accordingly, the values for B2MG, GUSB and POL2R were removed from the geNorm input file and the stability of GAPDH, HPRT, SDHA and TBP were re-assessed. When these four reference genes were evaluated in the absence of the most stable genes, the M values were under 1.5 (not shown), however, the threshold of 0.15 in the pairwise variation analysis was not achieved (far right bar in Figure 2b).

**Figure 3:**
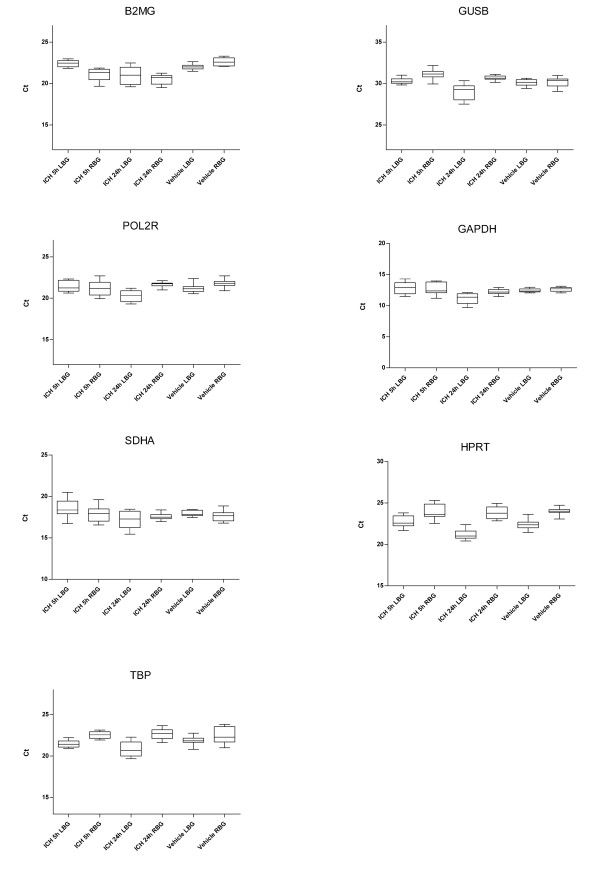
**Average real-time RT-PCR cycle threshold (Ct) values of candidate reference genes**. Data are displayed as average raw Ct (not yet normalised) values from individual groups of collagenase ICH and saline vehicle rats. The line in the box indicates the median, while the box represents the 25^th ^and 75^th ^percentile. Whiskers represent the maximum and minimum values.

**Figure 4:**
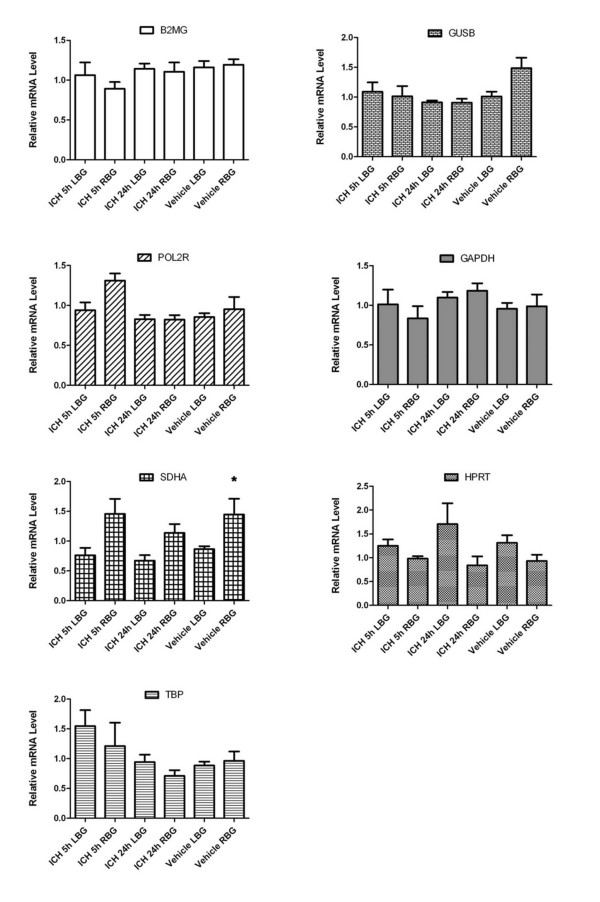
**Normalised mRNA levels of candidate reference genes**. The mRNA level of each group (5 hour and 24 hour collagenase ICH and saline vehicle rats) was normalised to the geometric mean of the expression of the four most stable reference genes determined by geNorm (B2MG, GUSB, POL2R and GAPDH). Each bar represents the normalised mean of triplicate measurements from 5 animals, ± SEM. Asterisk denotes statistical significance (*p *< 0.05) between ICH 24 hour LBG and vehicle RBG samples by one-way ANOVA.

Figure 3 shows the average raw (i.e. not yet normalised) Ct values of the collagenase ICH and saline vehicle rats for each candidate reference gene. The raw Ct values of GAPDH were fairly consistent between groups, while HPRT exhibited more variation. The collagenase ICH samples generally had more raw Ct variation than the saline vehicle samples. In Figure 4, the raw Ct values were normalised to the geometric mean of the four most stable reference genes determined by geNorm (B2MG, GUSB, POL2R and GAPDH), in order to obtain the relative mRNA level of each reference gene. One-way ANOVA revealed statistically significant differences in relative mRNA level within the SDHA group.

### TRPM2 mRNA Level Normalised to Multiple Reference Genes

**Figure 5:**
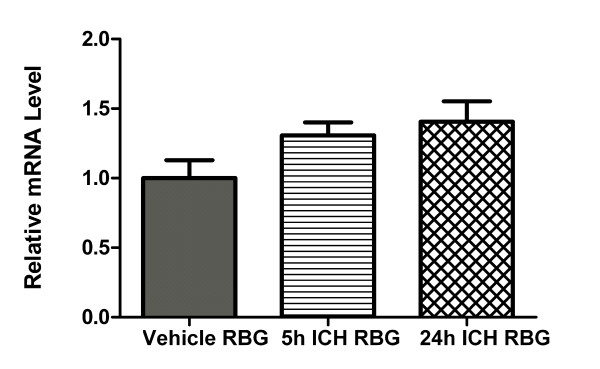
**Normalised mRNA level of TRPM2 relative to multiple reference genes**. TRPM2 mRNA level in the perihematomal brain region (RBG) of collagenase-induced ICH animals was compared to saline vehicle controls, at 5 h and 24 h survival times. The most stable reference genes determined by geNorm were used for normalisation in each group (5 h: GAPDH, HPRT, POL2R, SDHA; 24 h: B2MG, GUSB, POL2R). Bars represent mean of triplicate measurements from 5 animals, ± SEM.

The qBasePlus program was used to calculate the normalised mRNA level of TRPM2 in the perihematomal brain region at 5 h and 24 h post-ICH, relative to the most stable reference genes for each time point as determined by geNorm (5 h: GAPDH, HPRT, POL2R and SDHA; 24 h: B2MG, GUSB and POL2R). qBasePlus utilises a modified version of the 2^-ΔΔCt ^method of relative expression analysis [37] that takes into account multiple reference genes and gene-specific amplification efficiencies [38]. There was no significant difference in mean TRPM2 transcript levels between collagenase ICH and saline vehicle animals (Figure 5).

## Discussion

In this study we have evaluated reference genes for use as real-time RT-PCR normalising factors in collagenase-induced ICH. Based on our results, we conclude that use of a single normalisation reference gene is potentially hazardous, and suggest a panel of reference genes for more accurate transcript quantification.

Real-time RT-PCR is a robust and sensitive technique for quantifying mRNA transcripts, and constitutes a powerful tool for increasing our understanding of the genomic response to ICH. It requires an appropriate normalisation strategy to control for error, the most common being the use of one or more endogenous reference genes [39]. A reference gene should be expressed at a stable level regardless of the experimental context, however, the expression of commonly used reference genes has been shown to vary considerably. Normalisation of real-time RT-PCR data using a single, non-validated reference gene may lead to inaccurate biological conclusions, and previous studies have highlighted the need to validate reference genes for each new experimental condition [7,9,15].

Our previous study [40] identified suitable reference genes for normalising real-time RT-PCR data in the rat cerebral cortex and hippocampus following traumatic brain injury (TBI). The present study focuses on a different neurological disorder with a different animal model, brain regions and survival times to our previous study. Our results demonstrate that the panels of stable reference genes in ICH are not the same as those identified in our previous TBI reference gene study [40], consistent with the recommendation that reference gene validation be carried out whenever a new experimental variable is introduced [9]. Reference gene validation was thus a crucial prerequisite to gene expression analysis in ICH, and the current study has identified the appropriate panel of reference genes for further studies.

Alternative normalisation strategies include the use of exogenous standards and normalisation to total RNA. Several groups have reported reliable normalisation with exogenous standards [41-43]. A known amount of exogenous standard can be incorporated into the RNA extraction process, which will then be affected by the same experimental error as the RNA of interest [44]. However, exogenous standards can be difficult to produce and are subject to degradation, and do not control for differences in quality of RNA template [17]. Another approach is to normalise to total RNA mass, which consists predominantly of ribosomal RNA (rRNA) molecules. However, it has been shown that rRNA content is not always an accurate predictor of the mRNA fraction [16]. Furthermore, normalisation to total RNA does not control for errors introduced during reverse transcription or PCR [44].

To our knowledge, this is the only study which has validated reference genes for use as normalising factors in the collagenase model of ICH in rats. We quantified the transcript level of seven candidate reference genes (B2MG, GUSB, GAPDH, HPRT, POL2R, SDHA and TBP) and our gene of interest, TRPM2, in the perihematomal and matching contralateral regions in rats 5 and 24 hours following collagenase-induced ICH, as well as in saline vehicle controls. Initially, data regarding TRPM2 transcript level in the perihematomal region were normalised to individual reference genes using the relative standard curve method of analysis [30]. At the 24 h survival time point, when TRPM2 data were normalised to either GUSB or HPRT, significant differences were found. However, no differences were found when normalised to other commonly-used reference genes. There was considerable variation between samples when data were analysed in this way (Figure 1). However, with normalisation to a reference gene panel far more consistent results were obtained (Figure 5). Had we used HPRT or GUSB as a single reference gene for normalisation, it is likely that we would have drawn erroneous conclusions about our data. These results highlight the importance of validating the stability of reference genes to be used as normalising factors in real-time RT-PCR studies.

When analysing all ICH and saline vehicle samples together, the most stable reference genes were B2MG and GUSB, with four reference genes recommended as the optimal number for accurate normalisation. The inclusion of additional reference genes further decreases the value of V, even when all seven genes are used for normalisation (V_6/7_). However, once the threshold value of 0.15 has been reached, additional reference genes do not significantly increase the reliability of normalisation [16]. Although the observed differences in reference gene stability were subtle, this does not imply that any reference gene combination would be appropriate. To illustrate this point, removing the 3 most stable reference genes (B2MG, GUSB and POL2R) from our geNorm input file showed that GAPDH, HPRT, SDHA and TBP assessed together do not meet the proposed cut-off value of V (0.15). Therefore, the most stable four genes, as determined by geNorm, should be used.

We also compared the stability of the candidate reference genes at individual time points following collagenase-induced ICH (5 and 24 hours). The most stable genes at 5 hours (GAPDH and HPRT) were different to those at 24 hours (B2MG and GUSB). Intriguingly, B2MG and GUSB were among the least stable genes in the 5 hour group, while GAPDH and HPRT were ranked 4^th ^and 6^th^, respectively, at 24 hours post-ICH. These results clearly demonstrate that stability of a candidate reference gene at one time point does not necessarily confer stability at another. Therefore, it is likely that the use of a single reference gene would be inadequate for normalising real-time RT-PCR data from different survival times of ICH. This is relevant because, as discussed, previous studies utilising real-time RT-PCR in rodent models of ICH generally have used one reference gene for normalisation without including a validation protocol. For example, HPRT was one of the most variably expressed genes in our study, but has been used as a single normalising factor in other studies quantifying mRNAs of interest over a time course of ICH [21,22]. It is possible that studies using a single, non-validated reference gene for normalisation could fail to detect small changes in the mRNA species of interest [14], or erroneously conclude that changes have occurred [15]. Indeed, another study from our laboratory [40] demonstrated that the mRNA level of our gene of interest varied significantly when normalised to individual reference genes, but was far more reliable when a panel of validated reference genes were used for normalisation.

The candidate reference genes in the present study also showed differences in stability between the perihematomal brain and the uninjured contralateral region. As might be expected, differences in gene stability were most marked in the perihaematomal region. A cascade of pathological processes including excitotoxicity, oedema and inflammation are initiated by ICH [18], all of which may impact reference gene expression.

## Conclusions

We have evaluated the expression stability of seven candidate reference genes following collagenase-induced ICH in rats. We have identified B2MG, GUSB, POL2R and GAPDH as an appropriate panel of reference genes to be used in the acute phase (5 and 24 hours) of experimental ICH. When analysed in subsets of these samples (according to survival time and brain region), variations in the stability of the reference genes were revealed. Our gene of interest, TRPM2, was inconsistently elevated following injury when normalised to individual reference genes. However, when normalised to a panel of the most stable genes, no significant differences were seen. These results emphasise the importance of identifying and validating suitable reference genes to prevent erroneous conclusions. The results of the present study will enable more accurate normalisation of real-time RT-PCR data at 5 and 24 hour time points following collagenase-induced ICH.

## Methods

### Collagenase-induced ICH

All animal protocols were approved by the Institute of Medical and Veterinary Science and the University of Adelaide Animal Ethics committees and were conformed to guidelines issued by the Australian National Health and Medical Research Council.

Adult male Sprague-Dawley rats (n = 15) weighing 300-340 g were used in the study, and obtained four days prior to surgery to ameliorate any acute stress-induced changes in gene expression. The study used collagenase to induce intracerebral hemorrhage [45]. Briefly, animals were anesthetized using isoflurane (1.5 - 2.0%) in a 30:70 mix of oxygen and nitrogen via a nose cone and placed in a Kopf stereotaxic frame. A rectal thermometer was inserted and temperature maintained between 36.5 - 37.5°C with a thermostatically-controlled heat pad. The scalp was shaved and cleaned, and bupivacaine instilled. A midline scalp incision was made, the skull exposed and a burrhole drilled 0.7 mm anterior and 3.0 mm lateral to bregma. Using a syringe driver (Harvard Instruments), 0.2 U type VII bacterial collagenase (Sigma C0773) in 2 μL normal saline, or saline alone ('vehicle') was infused over 4 minutes, via a 30 G needle, into the centre of the striatum (6.0 mm anterior to bregma). The needle was left in place for five minutes, and then withdrawn slowly. The hole was sealed with bone wax and the scalp wound closed with wound clips after irrigation with bupivacaine.

Animals recovered in their home cage in a climate- and light-controlled environment with free access to food and water. Animals were killed by decapitation under deep isoflurane anesthesia either at 5 or 24 hours post-surgery. The brain was quickly extracted and a 4 mm thick slice taken of cortex and basal ganglia, incorporating the injection site at the centre and the bulk of the perihematomal brain. This was bisected into right and left hemispheres and immediately snap-frozen for RNA extraction.

### RNA Extraction

Total RNA was extracted from the left and right striatum with overlying cortex ('LBG' (basal ganglia) and RBG, respectively) of collagenase ICH rats (n = 5 from each time point) and saline vehicle rats (n = 5) using the RNeasy Lipid Tissue kit (Qiagen, Doncaster, Australia) according to the manufacturer's instructions. Fifty mg tissue was used in each RNA extraction, which included an on-column DNase treatment step (Qiagen). RNA was quantified by UV spectrometry using the Nanophotometer (Implen, Australia) to measure absorbance at 230, 260 and 280 nm. RNA integrity was evaluated using the Agilent Bioanalyzer RNA 6000 Nano Chip (Series II) kit.

### Reverse Transcription

Complementary DNA was synthesised using the SuperScript III Reverse Transcription kit (Invitrogen, Mt Waverley, Australia). Two μg total RNA was added to 250 ng random hexamers (Geneworks, Adelaide, Australia), 1 mM each dNTP (Invitrogen) and nuclease-free water to 13 μL. Reactions were heated to 65°C for 5 minutes then immediately placed on ice for 1 minute. To each tube, 4.75 μL 5× First Strand Buffer, 1 μL RNase OUT (Invitrogen), 0.02 M dithiothreitol and 200 units SuperScript III reverse transcriptase were added. Reactions containing nuclease-free water in place of enzyme served as negative controls. Reactions were incubated at 25°C for 5 minutes, 55°C for 60 minutes and 70°C for 15 minutes. cDNA was diluted to 10 ng/μL with nuclease-free water and stored at -20°C.

### Real-time PCR

The candidate reference genes used in this study were: GAPDH, B2MG, POL2R, TBP, HPRT, SDHA and GUSB. Primer sequences for reference genes have been described elsewhere [40]. TRPM2 primer sequences were (5' - 3'): forward, GAAGGAAAGAGGGGGTGTG and reverse, CATTGGTGATGGCGTTGTAG [46], with a product size of 101 base pairs. Real-time RT-PCR amplifications for reference genes and TRPM2 were carried out using 10 μL 2× Invitrogen Platinum SYBR Green SuperMix-UDG, 300 nM forward and reverse primers (400 nM for POL2R and GAPDH), 1 μL cDNA and nuclease-free water in a total volume of 20 μL. One hundred mM MgCl_2 _(Invitrogen) was added to POL2R reactions. A set of standards was included in each run, comprising five-fold serial dilutions made from aliquots of pooled cDNA, derived from an RNA pool of all samples. Serial dilutions contained the following amounts of cDNA: 50 ng, 10 ng, 2 ng and 0.4 ng. The standard series encompassed the unknown cDNA concentrations. Amplification was carried out in a Rotor-Gene 3000 (Corbett Research, Mortlake, Australia) with an initial UDG incubation of 50°C for 2 minutes, initial denaturation of 95°C for 2 minutes, followed by 40 cycles of: 95°C for 15 sec denaturation, primer-specific annealing temperature for 15 sec (see [40]), and 72°C for 15 sec extension. TRPM2 annealing temperature was 60°C. Fluorescence data were collected during the extension step of each cycle. Specificity of amplicons were verified by melting curve analysis after 40 cycles (72°C to 95°C) and 2% agarose gel electrophoresis stained with ethidium bromide and visualised under UV light. All cDNA samples were run in triplicate. Negative controls containing water instead of cDNA were present in all runs. No-reverse transcriptase controls were included for each gene to test for genomic DNA amplification.

### Data Analysis

Standard curves made from serial dilutions of pooled cDNA were used to calculate PCR efficiency (E) using the formula: E (%) = (10^[-1/slope] ^- 1) × 100. Raw values of input RNA were determined for each sample from the standard curve using the Corbett Rotor-Gene 6 software. The relative standard curve method [30] was applied to these raw quantities to calculate TRPM2 mRNA level relative to each of the seven reference genes individually. The cycle threshold (Ct) of an individual sample reflects the cycle at which a detectable number of PCR products have accumulated above background fluorescence [47]. Ct values were calculated from the standard curve, entered into the qBasePlus software [38] and used to generate an input file for geNorm v3.5 [16]. geNorm determined the most stable reference genes out of the panel of candidate genes using expression stability analysis by pairwise correlations. An expression stability measure, *M*, was assigned to each gene, which was used to rank candidate reference genes in order of stability. The most stable reference genes were determined in the following groups: all collagenase ICH and saline vehicle samples; 5 hour collagenase ICH and saline vehicle samples; 24 collagenase ICH and all saline vehicle samples; RBG only from all collagenase ICH and saline vehicle samples; LBG only from all collagenase ICH and saline vehicle samples. Normalised mRNA levels of each gene were calculated using qBasePlus once the most stable reference genes had been determined. Statistical analyses were carried out using unpaired Student's t-tests and one-way analysis of variance (ANOVA) with Newman-Keuls *post-hoc *tests.

## Authors' contributions

NLC carried out the reference gene study, performed the statistical analyses and drafted the manuscript. TJK carried out the animal surgery, and assisted with statistical analyses and drafting the manuscript. CVDH supervised the study, participated in study design and provided useful discussion. RV participated in study design and coordination and provided useful discussion. All authors read and approved the final manuscript.
